# Biocontrol Mechanism of *Bacillus thuringiensis* GBAC46 Against Diseases and Pests Caused by *Fusarium verticillioides* and *Spodoptera frugiperda*

**DOI:** 10.3390/biom15040519

**Published:** 2025-04-01

**Authors:** Zhao Liang, Qurban Ali, Huijun Wu, Qin Gu, Xin Liu, Houjun Sun, Xuewen Gao

**Affiliations:** 1 The Sanya Institute, Nanjing Agricultural University, Sanya 572024, China; 20240105@jaas.ac.cn (Z.L.); hjwu@njau.edu.cn (H.W.); guqin@njau.edu.cn (Q.G.); 2 Department of Plant Pathology, College of Plant Protection, Nanjing Agricultural University, Nanjing 210095, China; 3 Key Laboratory of Biology and Genetic Improvement of Sweet Potato, Xuzhou Institute of Agricultural Sciences in Jiangsu Xuhuai Area, Ministry of Agriculture, Xuzhou 221131, China; 20071004@jaas.ac.cn; 4 Department of Biology, College of Science, United Arab Emirates University, Al-Ain P.O. Box 15551, United Arab Emirates; rattarqurban@uaeu.ac.ae; 5 Institute of Food Safety and Nutrition, Jiangsu Academy of Agricultural Sciences, Nanjing 210014, China; xinliu@jaas.ac.cn

**Keywords:** *Bacillus thuringiensis*, *Fusarium verticillioides*, *Spodoptera frugiperda*, interaction relationship, biological control

## Abstract

*Bacillus thuringiensis* (*Bt*) is widely recognized as the most important microbial pesticide controlling various insect pests and diseases due to its insecticidal crystal proteins (ICPs) and antimicrobial metabolites. The current study investigates the biocontrol potential of *B. thuringiensis* GBAC46 against the fungal pathogen *Fusarium verticillioides* and the insect pest *Spodoptera frugiperda* through multiple mechanisms. Phenotypic experiments revealed that GBAC46 effectively inhibited *F. verticillioides* growth by inducing reactive oxygen species (ROS) accumulation and showed enhanced larvicidal activity against second instar *S. frugiperda* larvae. Pot experiments showed that feeding by *S. frugiperda* enhanced *F. verticillioides* infection in maize. The *Bt* strain GBAC46 effectively controlled both pests and diseases in greenhouse maize seedlings. Applying the *Bt* strain GBAC46 reduced feeding damage from *S. frugiperda*, decreased leaf yellowing and wilting caused by *F. verticillioides*, and improved growth indicators such as plant height, fresh weight, and dry weight. RT-qPCR results revealed that the *Bt* strain GBAC46 induced key defense genes in maize involved in activating salicylic acid, jasmonic acid, and ethylene pathways. The overall study demonstrated and confirmed the GBAC46 strain as a promising microbial agent for disease and pest management.

## 1. Introduction

Plants in nature inevitably face a wide range of biotic stresses throughout their growth and development [1]. Among these, pathogenic microorganisms and phytophagous pests are the most prevalent and significant stressors, posing serious threats to crop growth and global food security [2]. Pathogenic fungi and insect pests frequently share the same host plant, both causing significant damage to crops and plants. The interaction between phytopathogens and phytophagous pests on a common host can vary, ranging from independent effects to more complex interdependencies [3,4]. More commonly, this interaction can manifest either as an inhibitory relationship [5,6] or as a mutually reinforcing relationship [7]. Research has shown that plant pathogenic microorganisms can promote feeding, development, and egg-laying in phytophagous pests [8,9].

Plant diseases caused by pathogenic microorganisms can alter the food requirements [2] and feeding preferences of pests [10]. Conversely, research has demonstrated that plant pathogenic microorganisms can also negatively affect pest feeding behavior and development [11]. Pests may also either facilitate the infection of plants by pathogenic microorganisms [12] or inhibit such infections [13,14]. Furthermore, pests can serve as vectors for pathogen transmission [15]. Phytophagous pests and pathogenic microorganisms frequently co-exist on the same host plants, leading to concurrent outbreaks of pest infestations and diseases. The interactions among pathogens, pests, and plants are complex and have cumulative effects on plant growth and yield [16]. This complexity makes the simultaneous management of plant pests and diseases particularly challenging. Therefore, the identification and development of biopesticides that can effectively control both pests and diseases are crucial for the sustainable advancement of global agriculture and food security.

*Bacillus thuringiensis* (*Bt*), a Gram-positive, spore-forming soil bacterium, has emerged as one of the most successful biological agents (BCAs) in agriculture, primarily due to its production of parasporal crystal proteins (Cry or δ-endotoxins) [17,18]. Renowned as an insect pathogen, *Bt* insecticidal activity is attributed to these Cry produced during sporulation and other major toxins factors (such as Vip and Cyt toxins), which exhibited toxicity against a broad spectrum of insect species across various orders, including Coleoptera, Hymenoptera, Diptera, Homoptera, and Orthoptera. Additionally, *Bt* is effective against nematodes, mites, and protozoa [19,20].

In addition to its potent insecticidal toxins, numerous studies have revealed that *Bt* exhibits significant antifungal activity against various plant pathogenic fungi, such as *Fusarium* spp., *Sclerotium* spp., *Colletotrichum* spp., *Rhizoctonia* spp., and *Botrytis* spp. [21,22,23,24,25,26]. This activity is attributed to its ability to secrete cell wall-degrading enzymes, lipopeptide fengycin, volatile organic compounds (VOCs), and chitinase. *Bt* holds great potential as a biopesticide for the integrated management of both plant diseases and pests. *B. thuringiensis* GBAC46, isolated from rhizosphere soil of grassroots at an altitude of 3421 m in Qinghai-Tibet Plateau, has demonstrated cold-tolerant, plant growth-promoting properties in cold environments and the ability to suppress a wide range of plant pathogens [27]. These characteristics make it a promising candidate for the development of formulations aimed at sustainable agricultural approaches. Previous research revealed that GBAC46 encodes three novel Cry proteins (namely, Cry31Aa, Cry73Aa, and Cry40ORF), one of which exhibits nematicidal activity against the rice parasitic nematode *Aphelenchoides besseyi* [17]. These findings highlight the strain potential for dual biocontrol applications targeting both plant pests and diseases.

In this study, maize seedlings were utilized as a model plant to investigate the biocontrol efficacy of *Bt* strain GBAC46 against the fungal pathogen *F. verticillioides* and the insect pest *S. frugiperda*, as well as their simultaneous occurrence on the maize host plant. In addition to assessing its ability to control this pathogen and insects, the growth-promoting effects of GBAC46 on maize were evaluated along with the interaction between *F. verticillioides* and *S. frugiperda* during co-infection. The results indicated that the feeding activity of *S. frugiperda* significantly enhanced the susceptibility of maize *F. verticillioides* infection, leading to pronounced impacts on maize plant development. These findings underscore the importance of integrating entomological and pathological research to better understand the dynamics of pest and disease occurrence in agricultural ecosystems. Such an integrated approach is essential for devising more effective monitoring systems and developing crop protection approaches to managing the combined threats posed by pests and pathogens in sustainable agriculture.

## 2. Materials and Methods

### 2.1. Strains, Phytophagous Insects, and Plant Materials

The *B. thuringiensis* GBAC46 and NMTD81 were separately isolated from rhizosphere soil of grass roots and barley roots at altitudes exceeding 3000 m in the Qinghai-Tibet Plateau [27]. The two *Bt* strains and the well-known biological control strain *B. velezensis* FZB42 used in this study are listed in Table S1. The fungal strains *F. verticillioides* 7600, *F. oxysporum f.* sp. *lycopersici* 4287, *S. sclerotiorum* (Lib.), and *R. solani* AG-1 were used for the antifungal activity assay (Table S1). All fungal and bacterial strains were stored at −80 °C in the refrigerator (Me Ling) in 60% and 15% glycerol solution at Biological Control and Bacterial Molecular Biology Lab NJAU, China. All strains were precultured in Luria–Bertani medium (LB), and fungal strains were precultured in the potato dextrose agar (PDA) medium. The second instar larvae of *S. frugiperda* (KE YUN) were purchased from Taobao along with their feed. Maize variety (DiKa 517) was selected for in vitro and pot experiments conducted in greenhouse environments.

### 2.2. Antifungal Activity Assay

The antifungal activity of selected bacterial strains was assessed using a dual culture assay. Culture discs (0.6 cm) of various plant pathogenic fungi *F. verticillioides*, *F. oxysporum*, *S. sclerotiorum*, and *R. solani* were placed at the center of Petri plates containing PDA medium. The Petri plates were then sealed with parafilm and incubated at 25 °C for 2–3 days before inoculation with *Bt* strains. Following this, 5 µL of (OD_600_ = 10^6^ CFU/mL) an overnight culture of GBAC46, NMTD81, FZB42, and a negative control (LB medium) was inoculated 3 cm from the edge of the fungal growth on a sterilized filter paper disc. The plates were tightly closed with parafilm and incubated at 25 °C for an additional 5–7 days. The experiment was conducted in triplicate, with three replicates for each treatment.

### 2.3. Determination of Reactive Oxygen Species

To investigate the underlying mechanism by which the GBAC46 strain inhibits *F. verticillioides*, we determined the accumulation of reactive oxygen species (ROS) in *F. verticillioides* hyphae using the probe dichloro-dihydro-fluorescein diacetate (DCFH-DA). Fluorescent signals were detected using fluorescence microscopy (Thermo Fisher Scientific, Hanover Park, IL, USA). The hyphae were treated with the overnight culture supernatant of the GBAC46 strain for 12 h, while LB medium treatment served as a negative control. Following treatment, mycelium was washed 2–3 times with 10 mM sodium phosphate buffer (pH 7.4) and, subsequently, incubated with 10 µM DCFH-DA for 30 min at 25 °C. Fluorescence signals were then observed under a microscope (Olympus IX71, Tokyo, Japan), capturing both the protonated form (excitation at 405 nm) and the deprotonated form (excitation at 458 nm). The experiment was conducted in triplicates and repeated three times under the same conditions.

### 2.4. Insecticidal Activity Assay

The overnight culture of the GBAC46 strain was collected and washed three times with sterile water. The culture was then adjusted to prepare high-concentration and low-concentration suspensions (1.8 × 10^8^ CFU/mL and 2.59 × 10^6^ CFU/mL, respectively). These suspensions were subsequently mixed evenly with agar-containing feed for *S. frugiperda* at a ratio of 1:10 (mL/g), following the dissolution of the solid feed through heating and cooling. The prepared feed was then used to treat second instar larvae in sterile 24-well plates, with mixed feed and sterile water serving as the negative control. Larval mortality of the instar larva was assessed after 10 days of treatment, with the feed being replaced every 2 days. Each treatment group included three biological replicates, with 24 larvae per replicate.

### 2.5. Greenhouse Experiments

The biocontrol efficacy of the GBAC46 strain against *F. verticillioides* and *S. frugiperda* was evaluated through pot experiments conducted in a greenhouse setting. The experimental setup included eight treatments: *F. verticillioides*, *S. frugiperda*, and *F. verticillioides* + *S. frugiperda*, GBAC46, GBAC46 + *F. verticillioides*, GBAC46 + *S. frugiperda*, GBAC46 + *F. verticillioides* + *S. frugiperda*, and a negative control (sterile water CK). Second instar larvae of *S. frugiperda* and a spore suspension of *F. verticillioides* (10^6^ CFU/mL) were inoculated and sprayed onto maize seedlings 20 days post-planting. Two days later, an overnight culture of GBAC46 strain (OD_600nm_ = 1.0) was sprayed on the leaves of the treated maize seedlings. Each treatment included 10 potted plants with 3 biological replicates. Plant growth parameters, including fresh weight, dry weight, and seedling height, were recorded within 10–15 days post-treatment. Disease incidence and seedling growth health were monitored daily throughout the experiment periods.

### 2.6. Field Experiment and Determination of Fumonisins B1

Maize cultivar DiKa 517 was selected for a field experiment conducted in a continuous maize cropping system in Suzhou City, Anhui Province, China, from June to October 2021. Three experimental designs included three treatments: (1) Control 1 (CK1), involving no application of insecticides or biological agents; (2) Control 2 (CK2), consisting solely of chemical insecticide application; and (3) combined treatments with both insecticide and the GBAC46 biocontrol agent (BCAs). The chemical insecticide diphenylthiamethoxam was applied at a rate of 50 mL/acre. After applying chemical insecticides for 4 days, *Bt* strain GBAC46 suspension was applied, and both were sprayed by drones. Each treatment was replicated three times in a random field layout, with each replicate containing 150 maize plants. According to the area of the lesion, the degree of maize seedling blight disease on maize cobs was graded and quantified into different disease class numbers (0, 1, 3, 5, and 7). The disease index (DI) was calculated using the formula:

 DI=[∑(class number×number of maize cobs in each class number)]×100/(total number of maize cobs×the highest disease class number)

To assess fumonisin B1 (FB1) production in maize grains under various treatments, kernels were dried at 30 °C and ground into a powder, and subsampled (12 samples per replicate). The FB1 concentration (per gram of maize kernel tissue) was quantified using a Waters 1525 high-performance liquid chromatography (HPLC) system and liquid chromatography/mass spectrometry (LC-MS), following the method previously described [28].

### 2.7. Extraction of Total RNA and Expression Analysis by RT-qPCR

To assess the expression of key defense-related genes in maize seedlings (listed in Table S2), total RNA was extracted from the leaves of maize seedlings inoculated with GBAC46 culture suspension or *S. frugiperda* for 24 h. The extraction was performed using the plant extraction kit (Omega Bio-tek, Norcross, GA, USA), following the manufacturer’s protocol. The purity and concentration of the extracted total RNA were measured using a NanoDrop1000 (Thermo Scientific, Wilmington, DE, USA). The extracted RNA was reverse transcribed into cDNA using the HiScript II Q RT SuperMix Kit (Vazyme, Nanjing, China) according to the manufacturer’s instructions. RT-qPCR was then performed using SYBR Premix Ex Taq (Takara Biotechnology Co., Dalian, China) in a 7500 Fast Real-Time PCR Detection System (QuantStudio-6 Thermo Fisher Scientific, San Jose, CA, USA). The relative gene expression levels were calculated using the ΔΔCT method with the 7500 system SDS software 2.0 (Applied Biosystems), as described previously [29]. All expression data represent the averages of three independent biological replicates, and each is conducted in technical triplicates.

### 2.8. Statistical Analysis

All experiments were conducted using a completely randomized design. The experimental data were subjected to statistical analysis using SPSS statistical software 25.0, with means separated by Tukey’s HSD test at *p* ≤ 0.05 following ANOVA.

## 3. Results

### 3.1. Bt Strain GBAC46 Exhibits Significant Antifungal Activity

**Figure 1:**
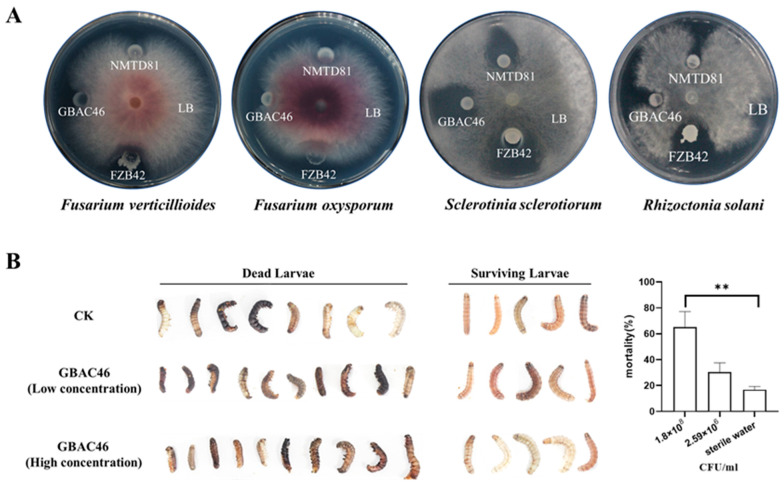
Determination and evaluation of GBAC46 strain biocontrol activity against plant pathogenic fungi and phytophagous pests. (**A**) Antifungal activity of bacterial cultures against several plant pathogenic fungi in the potato dextrose agar (PDA) medium. (**B**) Morphological differences and mortality rate of *Spodoptera frugiperda* larvae treated with GBAC46 strain. The phenotypic experiments were conducted in triplicate, yielding consistent results. Asterisks (**) indicate significant differences in the data.

The broad-spectrum antifungal activity of GBAC46, NMTD81, and FZB42 were assessed in dual plate methods. The broad-spectrum antifungal results demonstrated that all bacterial strains significantly inhibited the growth of *F. verticillioides*, *F. oxysporum*, *S. sclerotiorun*, and *R. solani* compared to the control (Figure 1A). Compared to well-known biological control agents FZB42, GBAC46 showed a similar inhibitor effect against *F. verticillioides*, *F. oxysporum*, *S. sclerotiorun*, and *R. solani*, highlighting the considerable biocontrol potential.

### 3.2. Insecticidal Activity of Bt Strain GBAC46 Against S. frugiperda

In our previous study, three unique crystal proteins (namely, Cry31Aa, Cry73Aa, and Cry40ORF) were identified from *Bt* strain GBAC46 through protein-nucleic acid alignment [17], indicating that the GBAC46 could be a promising candidate for insecticidal applications. Therefore, the insecticidal efficacy of this GBAC46 was evaluated in sterile 24-well plates against *S. frugiperda*. The findings revealed that the GBAC46 strain significantly increased the second instar killing mortality rate to 65.10% at a concentration of 1.8 × 10^8^ CFU/mL, compared to the control (CK) (18.22%, *p* < 0.01). In contrast, no significant difference in mortality rate was observed between the low-concentration treatment (2.59 × 10^6^ CFU/mL) and the CK. Given that the GBAC46 strain encodes several Cry proteins, its insecticidal activity is likely attributed to these encoded Cry proteins (Figure 1B). In addition, the Cry proteins (Cry31Aa, Cry73Aa, and Cry40ORF) identified from the GBAC46 strains were tested individually. The results demonstrated that these novel single Cry proteins exhibited no significant morality effect on *S. frugiperda.* The overall results showed that GBAC46 may also synthesize other toxins due to the synergistic action of its cry proteins, which requires further research.

### 3.3. Reactive Oxygen Species Assays

**Figure 2:**
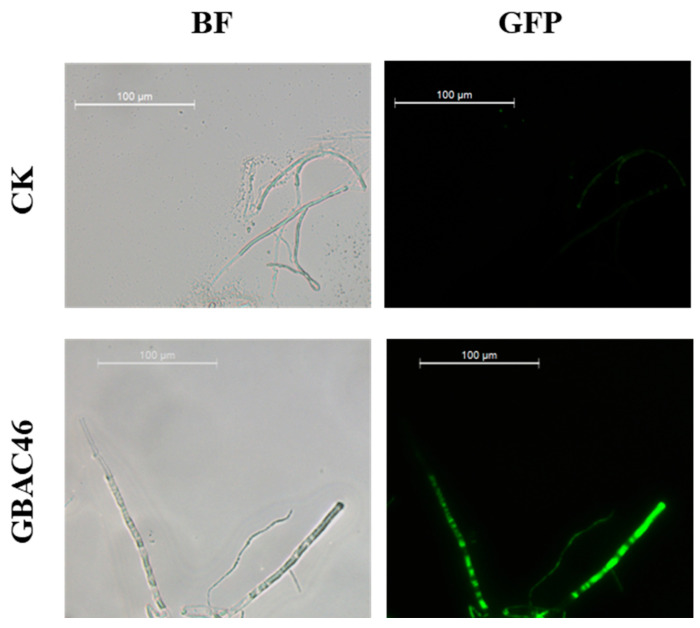
Effect of the GBAC46 strain overnight culture supernatant on the production of reactive oxygen species (ROS) in *F. verticillioides* hyphae under the green and white light fluorescence. Using DCFH-DA staining, ROS in the mycelia after different treatments were observed. The scale bar represents 100 µm. CK refers to LB medium treatment for 12 h, and GBAC46 means overnight culture supernatant treatment of *Bt* strain GBAC46 for 12 h. BF means bright field and GFP means green fluorescence field.

The impact of GBAC46’s culture supernatant on the burst and accumulation of ROS in fungal hyphae was detected using a DCFH-DA kit. The result showed that stronger green fluorescence was observed in hyphae treated with GBAC46’s culture supernatant compared to the negative control (Figure 2), indicating a large accumulation of ROS in the fungal mycelium.

### 3.4. Biocontrol Activity of GBAC46 Against Co-Occurring Diseases and Pests Caused by F. verticillioides and S. frugiperda

**Figure 3:**
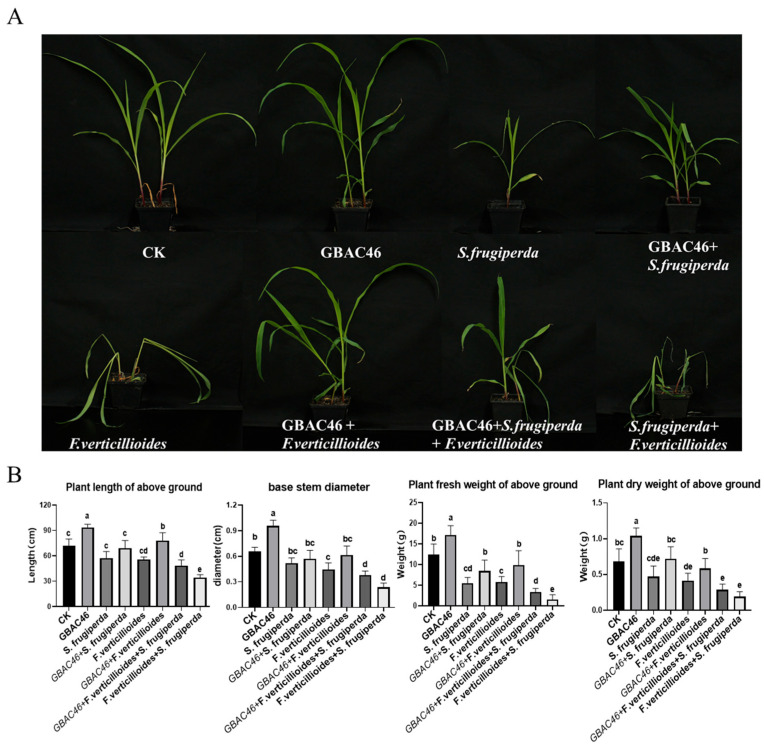
Evaluating the biocontrol activity of the GBAC46 strain against single and co-occurring diseases and pests caused by *F. verticillioides* and *S. frugiperda* and the effect of feeding by *S. frugiperda* on infection of *F. verticillioides* through pot experiments. (**A**) The growth rate of maize seedlings aboveground in different treatments. (**B**) Data collection and statistical analysis of plant height, plant fresh weight, plant dry weight, and base stem diameter of maize seedlings under varying treatments. The error bars on the graphs indicate the standard deviation of the mean (*n* = 10). The letters above the columns represent significant differences between treatments at *p* ≤ 0.05.

The inhibition of *F. verticillioides* growth and the insecticidal activity against *S. frugiperda* larvae suggest that GBAC46 possesses biocontrol potential properties against both pests and diseases. To further evaluate its efficacy, pot experiments were conducted to elucidate the GBAC46 strain’s ability to simultaneously prevent and control pests and diseases. When maize seedlings were inoculated only with *S. frugiperda*, the leaves were severely damaged by the larvae, resulting in a significant reduction in plant biomass, such as fresh and dry weight. However, in the treatment groups where plants were sprayed by overnight culture suspension of GBAC46, the extent of damage caused by *S. frugiperda* larvae was notably reduced. Additionally, plant growth parameters, including fresh, dry weight, and shoot length, increased significantly by 52.99%, 52.33%, and 19.92%, respectively (Figure 3A,B). These findings demonstrate that the GBAC46 exhibits strong biocontrol activity against *S. frugiperda*.

When maize seedlings were inoculated with *F. verticillioides* (at a concentration of 10^6^ CFU/mL spore suspension), they displayed typical symptoms of maize seedling blight, including stunted growth and leaf yellowing. In contrast, seedlings treated with an overnight culture suspension of GBAC46 showed significantly enhanced resistance to *F. verticillioides*, with notable improvements in growth and development. Specifically, plant height and fresh and dry weight increased by 39.81%, 69.96%, and 41.2%, respectively. Root growth also showed substantial improvements, with the fresh and dry weight of the roots increasing by 9.19% and 7.19% (Figure 3).

**Figure 4:**
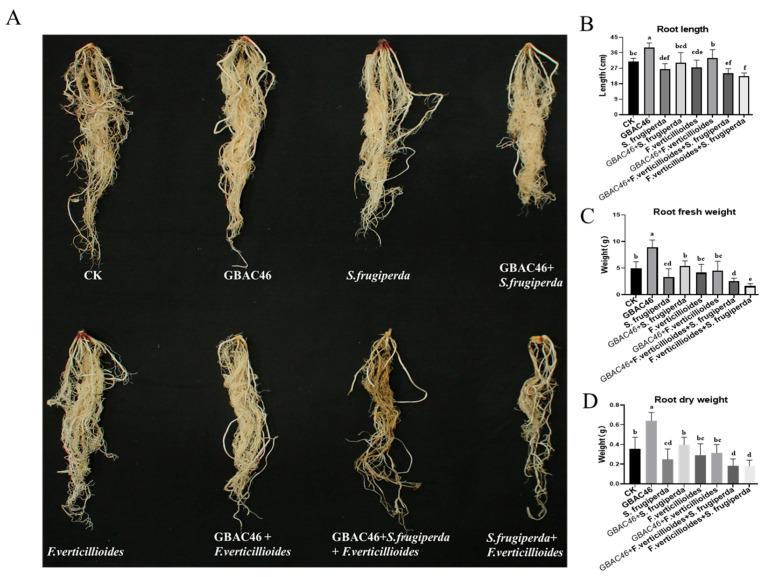
Testing the biocontrol activity of GBAC46 strain against *F. verticillioides* and *S. frugiperda* through maize root growth traits. (**A**) The growth status of maize roots in different treatments. (**B**–**D**) Data collection and statistical analysis of root length, root fresh weight, and root dry weight. The error bars on the graphs indicate the standard deviation of the mean (*n* = 10). The letters above the columns represent significant differences between treatments at *p* ≤ 0.05.

In addition, when maize seedlings were subjected to *S. frugiperda* and *F. verticillioides*, their growth was significantly impaired, as evidenced by reduced height, more severely atrophied leaves, and smaller root systems. Compared to seedlings exposed to *F. verticillioides* alone, those co-infected with both pathogens exhibited a marked decrease in root length fresh and dry weight by 18.05%, 61.36%, and 37.67%, respectively (Figure 4). This indicates that *S. frugiperda* feeding exacerbated *F. verticillioides* infection. Enhanced susceptibility can likely be attributed to the mechanical wounds caused by *S. frugiperda* feeding, which compromises the physical defenses of maize seedlings and facilitates easier penetration and parasitism by *F. verticillioides.* Notably, the symptoms induced by *F. verticillioides* and *S. frugiperda* were significantly alleviated upon application of the GBAC46 strain. Treated seedlings showed improvement in plant height, fresh and dry weight increased by 29.15%, 52.53%, and 34.15%, respectively. These findings indicated that *S. frugiperda* feeding, indeed, promotes *F. verticillioides* infection in maize seedlings and highlights the biocontrol potential of GBAC46 in effectively managing both the pest and pathogen.

### 3.5. Induced Systemic Resistance

**Figure 5:**
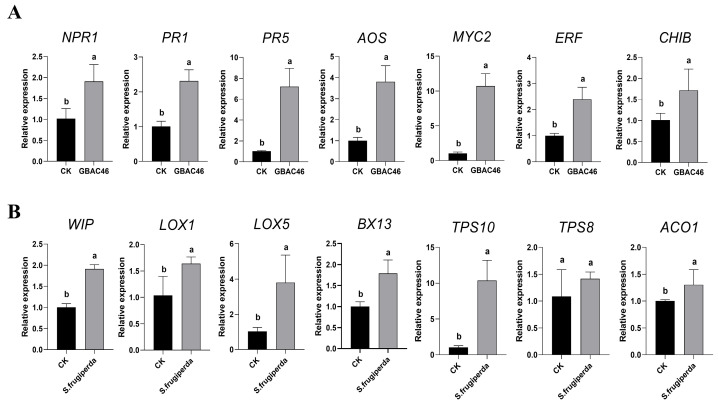
Profiling the expression levels of related genes in maize seedling systemic resistance response induced by GBAC46 strain and the feeding by *S. frugiperda by* RT-qPCR experiments. (**A**) The key genes *NPR1*, *PR1*, and *PR5* involved in the salicylic acid pathway, *AOS* and *MYC2* involved in the jasmonic acid pathway, and *ERF* and *CHIB* involved in the ethylene signaling pathway were selected to detect maize seedling systemic resistance response induced by the GBAC46 strain. (**B**) The key genes *LOX1*, *LOX5*, *BX13*, *TPS8*, *TPS10*, and *ACO1* related to primary and secondary metabolism and mechanical injury-induced protein (WIP) genes in maize were chosen to test maize seedling systemic resistance response induced by *S. frugiperda*. The error bars on the graphs indicate the standard deviation of the mean (*n* = 3). The letters above the columns represent significant differences between treatments at *p* ≤ 0.05.

The key genes in the salicylic acid pathway (*NPR1*, *PR1*, and *PR5*), the jasmonic acid pathway (*AOS* and *MYC2*), and the ethylene signaling pathway (*ERF* and *CHIB*) were selected for RT-qPCR experiments to assess relative expression changes 24 h post-treatment with the GBAC46 strain. The results indicated that the *PR5* and *MYC2* genes in maize exhibited the most significant responses to the GBAC46 strain, with great upregulation of 7.2-fold and 10.7-fold, respectively (Figure 5A). These findings confirm that the GBAC46 strain can induce systemic resistance in maize. Furthermore, upon herbivorous insect invasion, plants undergo morphological and structural changes, activating specific primary and secondary metabolic pathways [30,31,32,33]. To further investigate relative expression levels of key genes involved in benzoxazinoids (*Bxs*) synthesis (*BX13*), lipoxygenase synthesis (*LOX1* and *LOX5*), terpene synthesis (*TPS8* and *TPS10*), and wound-induced protein synthesis (*WIP*) genes were evaluated 24 h after *S. frugiperda* invasion. The results revealed significant upregulation of these genes, except for the *TPS8* (Figure 5B), indicating that the systemic defense response in maize had been effectively activated.

### 3.6. Effect of Biocontrol Potential of the GBAC46 Strain on Maize Seedlings

**Figure 6:**
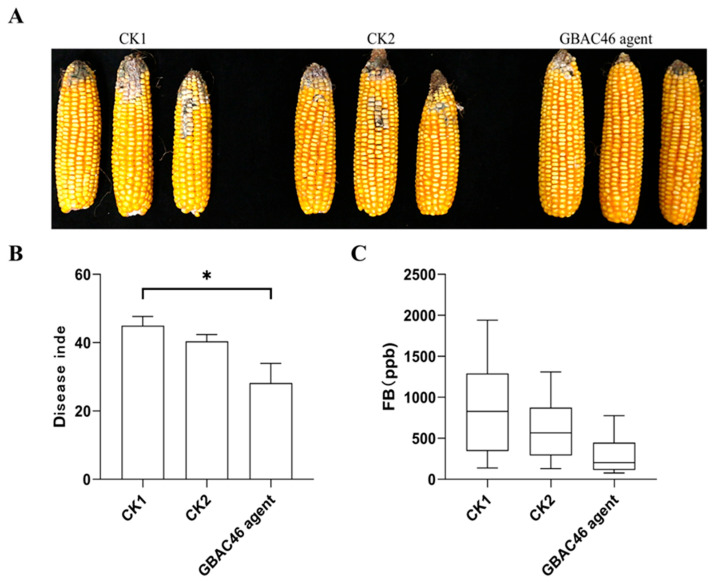
Characterizing the biocontrol activity of the GBAC46 agent against *F. verticillioides* through field experiments. (**A**) The biocontrol effect of GBAC46 strain on maize seedling blight. (**B**) Calculating the disease index of maize seedling blight by grading the symptoms. (**C**) Detecting the fumonisin B1 content in maize grains from different treatments. The error bars on the graphs indicate the standard deviation of the mean (*n* = 50), with three repetitions. Asterisks (*) indicate significant differences in the data.

To further assess the biocontrol potential of the GBAC46 strain against maize seedling blight, a field experiment was conducted. The disease incidence in maize cobs was graded and quantified, revealing that the CK1 group (untreated control) exhibited the highest disease index at 44.9 ± 2.1 %. In the CK2 group (treated with pesticides), the disease index was slightly lower at 40.3 ± 1.5% (Figure 6A). In contrast, maize affected by seedling blight and treated with the GBAC46 strain showed a significantly reduced disease index of 28.1 ± 4.7% (Figure 6B). Based on these results, the relative control efficacy of GBAC46 against maize seedling blight was determined at 37.4% compared to the CK1 treatment. Additionally, the fumonisin B1 content in maize grains from different treatments was analyzed. The grain treated with GBAC46 has a significantly lower fumonisins B1 content of 294.9 ± 25.9 ppb compared to the CK1 and CK2 groups (Figure 6C).

## 4. Discussion

In nature, herbivorous insects and pathogenic fungi often share the same host plant, leading to intricate interactions among these three entities [16,34]. This study investigated the interaction between *F. verticillioides* and *S. frugiperda* when both co-existed on the same maize seedlings. The findings revealed that *S. frugiperda* feeding facilitated *F. verticillioides* infection in maize, resulting in stunted seedling growth, yellowing, severe leaf atrophy, and a substantial negative impact on the overall growth and development of the plants (Figure 1). These data results are consistent with previously reported findings, for example, feeding damage of adult beetles provides entry ports for fungal colonization and promotes infection success by pathogenic fungi [35]. Similarly, the outcome of this study is likely attributable to the mechanical damage caused by *S. frugiperda* feeding, which compromises the maize plant’s physical defenses and enables easier pathogen entry. Previous studies have demonstrated that herbivorous insects can influence plant–pathogen interactions through both direct and indirect mechanisms. The direct effects often involve insects feeding on fungal spores, which can reduce the prevalence of pathogenic diseases [36]. Conversely, the insect feeding mechanically damages host plants, increasing their susceptibility to pathogen infection, as shown in this study.

Maize seedlings inoculated with *F. verticillioides* spores (10⁶ CFU/mL) exhibited leaf wilt symptoms. However, the co-application of *F. verticillioides* and *S. frugiperda* significantly heightened maize seedling blight severity, reducing plant height and biomass (Figure 3). This means phytophagous pests do facilitate the infection of plants by phytopathogens, consistent with the reported data [12,37]. Measurements of the plant height, fresh/dry aboveground weight, root length, and root biomass confirmed that *S. frugiperda* feeding intensified *F. verticillioides* infection. While insect feeding may activate plant defenses that inhibit pathogens [38], our results showed *S. frugiperda* feeding triggered a systemic defense response in maize. RT-qPCR analysis revealed significant upregulation of genes involved in metabolite synthesis, including terpenes (*TPS10*), benzoxazine compounds (*BX13*), lipoxygenase (*LOX1*, *LOX5*), and protease inhibitor defense proteins (*ACO1*), implying that a series of defense responses were activated, and these findings suggest that *S. frugiperda* feeding activates maize’s defense mechanisms (Figure 5). As many previous studies have reported, the feeding of herbivorous insects can induce plant defense responses [39,40].

The interaction between pathogens and herbivorous insects depends on temporal and spatial scales as systemic resistance in hosts takes time to develop [41]. This study aimed to align the invasion timelines of pathogens and herbivorous insects by simultaneously inoculating *F. verticillioides* and *S. frugiperda*, focusing on the effect of *F. verticillioides* on *S. frugiperda*. Pathogen-induced physiological changes in host plants can alter insect feeding behavior [42], egg production [43], and secondary metabolite production, which impacts insect growth [9,44]. Our findings revealed no significant differences in *S. frugiperda* larval length or weight, with or without *F. verticillioides*, suggesting minimal impact on larval growth and development. However, the potential effects on food intake, feeding preferences, and pupal formation in grasslands require further investigation.

Herbivorous insects and pathogenic fungi interact in complex ways, making pest and disease control challenging. *Bt* is known for its high toxicity to insects and nematodes due to ICP produced during sporulation [19,20,27,45]. It also inhibits plant pathogenic fungi through the secretion of active substances like chitinase, lipopeptides, volatile organic compounds (VOCs), and cell wall-degrading enzymes [46,47]. The *Bt* strain GBAC46 has shown strong toxicity against the rice parasitic nematode *Aphelenchoides besseyi* [17]. In this study, we discovered its antifungal activity against *F. verticillioides* and insecticidal effects against *S. frugiperda* larvae at high suspension concentrations, highlighting its biocontrol potential. A greenhouse experiment revealed that spraying GBAC46 significantly reduced maize seedling blight symptoms. Treated plants showed improved height, fresh weight, and dry weight compared to untreated controls; for example, plant height and fresh and dry weight increased by 39.81%, 69.96%, and 41.2%, respectively, confirming GBAC46’s efficacy in controlling *F. verticillioides* and associated diseases. *Bt* strains have been previously reported to have control efficacies against different plant pathogenic fungi such as *Fusarium* and *Sclerotium* spp. which is similar to our results [21,22,24,25].

Spraying GBAC46 overnight culture significantly reduced *S. frugiperda* feeding on maize seedlings, although the second instar larvae were not killed. *S. frugiperda* feeding, however, promoted *F. verticillioides* infection, causing stunted seedlings, yellowing, severe leaf atrophy, and impaired growth. Similarly, several other reports have also presented the promotion of herbivorous insects to infection of phytopathogens on hosts [12,48]. Co-treatment of maize seedlings with GBAC46, *F. verticillioides*, and *S. frugiperda* improved seedling growth, with notable increases in fresh and dry weights. *Bt* strains or crops play an important role in reducing the incidence of *Fusarium* and fumonisin levels on field crops [49]. In this study, a field experiment in Suzhou City assessed the biocontrol efficacy of GBAC46 against maize seedling blight. Although field control efficacy was moderate, the GBAC46 application achieved a 37.4% control rate compared to CK1. Fumonisin B1 levels in maize grains correlated with observed control efficacy, highlighting GBAC46 biocontrol potential (Figure 6).

The biocontrol mechanism of the GBAC46 strain against *F. verticillioides* was investigated from two key perspectives: direct inhibition of fungal growth and induction of systemic resistance (ISR) in plants. *Bacillus* spp. employs various antifungal strategies, including producing bioactive lipopeptides [50,51,52] and volatile organic compounds (VOCs) [42,53] and inducing excessive ROS in pathogens [54,55]. Excessive ROS disrupt cellular integrity by damaging DNA, proteins, and membranes, ultimately causing cell death [56,57]. In this study, fungal mycelium was exposed to GBAC46 culture supernatant for 12 h. Intense green fluorescence under a fluorescence microscope indicated ROS bursts and fungal cell death, aligning with previous findings [58,59] (Figure 2). Additionally, GBAC46 significantly upregulated genes involved in the salicylic acid pathway (*NPR1*, *PR1*, *PR5*), jasmonic acid pathway (*AOS*, *MYC2*), and ethylene signaling pathway (*ERF*, *CHIB*), with *PR5* and *MYC2* showing over sevenfold increases. These results demonstrate GBAC46’s dual action: it directly suppressing fungal pathogens and enhances plant systemic resistance, highlighting its potential for agricultural applications. Induced systemic resistance (ISR) serves as a crucial mechanism for enhancing plant defense against a wide array of pathogens and insect herbivores throughout the entire plant body [60].

## 5. Conclusions

In summary, this study demonstrates that the feeding activity of *S. frugiperda* facilitates the infection of *F. verticillioides* in maize seedlings. However, the biocontrol *Bt* strain GBAC46 effectively suppresses both the pest and the disease by inducing systemic resistance in plants. Understanding the interplay between plant diseases, pests, and environmental factors is crucial. Investigating these interactions can provide valuable insights for developing integrated prevention and control strategies, ultimately improving the management of pest and disease outbreaks. Finally, it is worth mentioning that there are still many biocontrol mechanisms of the *Bt* strain GBAC46 that remain unclear. For example, we have demonstrated that this strain can induce systemic resistance in maize, but it is not clear which metabolites play a specific role. In addition, the toxins against larvae of *S. frugiperda* from this strain are also unknown. These all require further research in the future.

## Data Availability

The data generated during this experiment are explained in detail in this manuscript.

## Supplementary Materials

The following supporting information can be downloaded at: https://www.mdpi.com/article/10.3390/biom15040519/s1, Table S1: List strains used in this study, Table S2: Related genes for RT-qPCR and its primers used in this study.

## References

[1] Miller R.N., Costa Alves G.S., Van Sluys M.A. (2017). Plant immunity: Unravelling the complexity of plant responses to biotic stresses. Ann. Bot..

[2] Ali Q., Ali M., Jing H., Hussain A., Manghwar H., Ali M., Raza W., Mundra S. (2024). Power of plant microbiome: A sustainable approach for agricultural resilience. Plant Stress.

[3] Franco F.P., Moura D.S., Vivanco J.M., Silva-Filho M.C. (2017). Plant-insect-pathogen interactions: A naturally complex ménage à trois. Curr. Opin. Microbiol..

[4] Willsey T., Chatterton S., Cárcamo H. (2017). Interactions of root-feeding insects with fungal and oomycete plant pathogens. Front. Plant Sci..

[5] Nicoletti R., Becchimanzi A. (2022). Ecological and molecular interactions between insects and fungi. Microorganisms.

[6] Rayamajhi M.B., Van T.K., Pratt P.D., Center T.D. (2006). Interactive association between *Puccinia psidii* and *Oxyops vitiosa*, two introduced natural enemies of *Melaleuca quinquenervia* in Florida. Biol. Control..

[7] Ariss J.J., Rhodes L.H., Sulc R.M., Hammond R.B. (2007). Potato leafhopper injury and fusarium crown rot effects on three alfalfa populations. Crop Sci..

[8] Friedli J., Bacher S. (2001). Mutualistic interaction between a weevil and a rust fungus, two parasites of the weed Cirsium arvense. Oecologia.

[9] Mondy N., Corio-Costet M.F. (2004). Feeding insects with a phytopathogenic fungus influences their diapause and population dynamics. Ecol. Entomol..

[10] Shapiro L., De Moraes C.M., Stephenson A.G., Mescher M.C. (2012). Pathogen effects on vegetative and floral odors mediate vector attraction and host exposure in a complex pathosystem. Ecol. Lett..

[11] Tack A.J.M., Gripenberg S., Roslin T. (2012). Cross-kingdom interactions matter: Fungal-mediated interactions structure an insect community on oak. Ecol. Lett..

[12] Kurtz B., Karlovsky P., Vidal S. (2010). Interaction between western corn rootworm (Coleoptera: Chrysomelidae) larvae and root-infecting *Fusarium verticillioides*. Environ. Entomol..

[13] Eyles A., Chorbadjian R., Wallis C., Hansen R., Cipollini D., Herms D., Bonello P. (2007). Cross induction of systemic induced resistance between an insect and a fungal pathogen in Austrian pine over a fertility gradient. Oecologia.

[14] Yang J.W., Yi H.S., Kim H., Lee B., Lee S., Ghim S.Y., Ryu C.M. (2011). Whitefly infestation of pepper plants elicits defense responses against bacterial pathogens in leaves and roots and changes the below-ground microflora. J. Ecol..

[15] Islam W., Noman A., Naveed H., Alamri S.A., Hashem M., Huang Z.Q., Chen H.Y.H. (2020). Plant insect vector-virus interactions under environmental change. Sci. Total Environ..

[16] Li Y., Duan T., Li Y. (2021). Research progress in the interactions of fungal pathogens and insect pests during host plant colonization. J. Plant Dis. Prot..

[17] Liang Z., Ali Q., Wang Y., Mu G., Kan X., Ren Y., Manghwar H., Gu Q., Wu H., Gao X. (2022). Toxicity of *Bacillus thuringiensis* strains derived from the novel crystal protein Cry31Aa with high nematicidal activity against rice parasitic nematode *Aphelenchoides besseyi*. Int. J. Mol. Sci..

[18] Peng Q., Yu Q., Song F. (2019). Expression of *cry* genes in *Bacillus thuringiensis* biotechnology. Appl. Microbiol. Biotechnol..

[19] Wan L.T., Lin J., Du H.W., Zhang Y.L., Bravo A., Soberon M., Sun M., Peng D.H. (2019). *Bacillus thuringiensis* targets the host intestinal epithelial junctions for successful infection of *Caenorhabditis elegans*. Environ. Microbiol..

[20] Xiao Y., Wu K. (2019). Recent progress on the interaction between insects and *Bacillus thuringiensis* crops. Philos. Trans. R. Soc. Lond. B Biol. Sci..

[21] Akram W., Mahboob A., Javed A.A. (2013). *Bacillus thuringiensis* strain 199 can induce systemic resistance in tomato against *Fusarium* wilt. Eur. J. Microbiol. Immunol..

[22] Reyes-Ramírez A., Escudero-Abarca B.I., Aguilar-Uscanga G., Hayward-Jones P.M., Barboza-Corona J.E. (2004). Antifungal activity of *Bacillus thuringiensis* chitinase and its potential for the biocontrol of phytopathogenic fungi in soybean seeds. J. Food Sci..

[23] Sadfi N., Cherif M., Fliss I., Boudabbous A., Antoun H. (2001). Evaluation of bacterial isolates from salty soils and *Bacillus thuringiensis* strains for the biocontrol of *Fusarium* dry rot of potato tubers. J. Plant Pathol..

[24] Shrestha A., Sultana R., Chae J.C., Kim K., Lee K.J. (2015). *Bacillus thuringiensis* C25 which is rich in cell wall degrading enzymes efficiently controls lettuce drop caused by *Sclerotinia minor*. Eur. J. Plant Pathol..

[25] Tang Y., Zou J., Zhang L., Li Z., Ma C., Ma N. (2012). Anti-fungi activities of *Bacillus thuringiensis* H3 chitinase and immobilized chitinase particles and their effects on rice seedling defensive enzymes. J. Nanosci. Nanotechnol..

[26] Zheng M., Shi J., Shi J., Wang Q., Li Y. (2013). Antimicrobial effects of volatiles produced by two antagonistic *Bacillus* strains on the an-thracnose pathogen in *postharvest mangos*. Biol. Control.

[27] Wu H.J., Gu Q., Xie Y.L., Lou Z.Y., Xue P.Q., Fang L., Yu C.J., Jia D.D., Huang G.C., Zhu B.C. (2019). Cold-adapted *Bacilli* isolated from the Qinghai-Tibetan Plateau are able to promote plant growth in extreme environments. Environ. Microbiol..

[28] Mirocha C.J., Kolaczkowski E., Xie W., Yu H., Jelen H. (1998). Analysis of deoxynivalenol and its derivatives (batch and single kernel) using gas chromatography/mass spectrometry. J. Agric. Food Chem..

[29] Liang Z., Qiao J.Q., Li P.P., Zhang L.L., Qiao Z.X., Lin L., Yu C.J., Yang Y., Zubair M., Gu Q. (2020). A novel Rap-Phr system in *Bacillus velezensis* NAU-B3 regulates surfactin production and sporulation via interaction with ComA. Appl. Microbiol. Biotechnol..

[30] Hilker M., Meiners T. (2010). How do plants “notice” attack by herbivorous arthropods?. Biol. Rev..

[31] Howe G.A., Jander G. (2008). Plant immunity to insect herbivores. Annu. Rev. Plant Biol..

[32] Kessler A., Baldwin I.T. (2002). Plant responses to insect herbivory: The emerging molecular analysis. Annu. Rev. Plant Biol..

[33] Mithofer A., Boland W. (2012). Plant defense against herbivores: Chemical aspects. Annu. Rev. Plant Biol..

[34] Bamisile B.S., Afolabi O.G., Siddiqui J.A., Xu Y. (2023). Endophytic insect pathogenic fungi-host plant-herbivore mutualism: Elucidating the mechanisms involved in the tripartite interactions. World J. Microbiol. Biotechnol..

[35] Gossner M.M., Beenken L., Arend K., Begerow D., Peršoh D. (2021). Insect herbivory facilitates the establishment of an invasive plant pathogen. ISME Commun..

[36] English-Loeb G., Norton A.P., Gadoury D.M., Seem R.C., Wilcox W.F. (1999). Control of powdery mildew in wild and cultivated grapes by a tydeid mite. Biol. Control..

[37] Hao Z.P., Sheng L., Feng Z.B., Fei W.X., Hou S.M. (2024). Aphids may facilitate the spread of Sclerotinia Stem Rot in Oilseed Rape by Carrying and Depositing Ascospores. J Fungi.

[38] Medeiros A.H., Franco F.P., Matos J.L., de Castro P.A., Santos-Silva L.K., Henrique-Silva F., Goldman G.H., Moura D.S., Silva-Filho M.C. (2012). Sugarwin: A sugarcane insect-induced gene with antipathogenic activity. Mol. Plant Microbe Interact..

[39] Guo J., Guo J., He K., Bai S., Zhang T., Zhao J., Wang Z. (2017). Physiological responses induced by *Ostrinia furnacalis* (Lepidoptera: Crambidae) feeding in Maize and their effects on *O. furnacalis* performance. J. Econ. Entomol..

[40] Ji M., Bui H., Ramirez R.A., Clark R.M. (2022). Concerted cis and trans effects underpin heightened defense gene expression in multi-herbivore-resistant maize lines. Plant J..

[41] Tack A.J.M., Dicke M. (2013). Plant pathogens structure arthropod communities across multiple spatial and temporal scales. Funct. Ecol..

[42] Rostas M., Simon M., Hilker M. (2003). Ecological cross-effects of induced plant responses towards herbivores and phytopathogenic fungi. Basic. Appl. Ecol..

[43] Ueda H., Kugimiya S., Tabata J., Kitamoto H., Mitsuhara I. (2019). Accumulation of salicylic acid in tomato plants under biological stress affects oviposition preference of *Bemisia tabaci*. J. Plant Interact..

[44] Hatcher P.E., Paul N.D., Ayres P.G., Whittaker J.B. (1994). The effect of a foliar disease (rust) on the development of *Gastrophysa-viridula* (Coleoptera, chrysomelidae). Ecol. Entomol..

[45] Guo S.X., Liu M., Peng D.H., Ji S.S., Wang P.X., Yu Z.N., Sun M. (2008). New strategy for isolating novel nematicidal crystal protein genes from *Bacillus thuringiensis* strain YBT-1518. Appl. Environ. Microb..

[46] Ali Q., Khan A.R., Tao S., Rajer F.U., Ayaz M., Abro M.A., Gu Q., Wu H., Kuptsov V., Kolomiets E. (2023). Broad-spectrum antagonistic potential of *Bacillus* spp. volatiles against *Rhizoctonia solani* and *Xanthomonas oryzae* pv. oryzae. Physiol. Plant..

[47] Jouzani G.S., Valijanian E., Sharafi R. (2017). *Bacillus thuringiensis*: A successful insecticide with new environmental features and tidings. Appl. Microbiol. Biotechnol..

[48] Ostry V., Ovesna J., Skarkova J., Pouchova V., Ruprich J. (2010). A review on comparative data concerning *Fusarium* mycotoxins in *Bt* maize and non-*Bt* isogenic maize. Mycotoxin Res..

[49] Barroso V.M., Rocha L.O., Reis T.A., Reis G.M., Duarte A.P., Michelotto M.D., Correa B. (2017). *Fusarium verticillioides* and fumonisin contamination in *Bt* and non-*Bt* maize cultivated in Brazil. Mycotoxin Res..

[50] Cawoy H., Debois D., Franzil L., De Pauw E., Thonart P., Ongena M. (2015). Lipopeptides as main ingredients for inhibition of fungal phytopathogens by *Bacillus subtilis*/*amyloliquefaciens*. Microb. Biotechnol..

[51] Farzand A., Moosa A., Zubair M., Khan A.R., Massawe V.C., Tahir H.A.S., Sheikh T.M.M., Ayaz M., Gao X. (2019). Suppression of *Sclerotinia sclerotiorum* by the induction of systemic resistance and regulation of antioxidant pathways in tomato using fengycin produced by *Bacillus amyloliquefaciens* FZB42. Biomolecules.

[52] Ongena M., Jacques P. (2008). *Bacillus* lipopeptides: Versatile weapons for plant disease biocontrol. Trends Microbiol..

[53] Gao H., Li P., Xu X., Zeng Q., Guan W. (2018). Research on volatile organic compounds from *Bacillus subtilis* CF-3: Biocontrol effects on fruit fungal pathogens and dynamic changes during fermentation. Front. Microbiol..

[54] Zhang L., Sun C. (2018). Fengycins, cyclic lipopeptides from marine *Bacillus subtilis* strains, kill the plant-pathogenic fungus *Magnaporthe grisea* by inducing reactive oxygen species production and chromatin condensation. Appl. Environ. Microbiol..

[55] Wang Y., Zhang C., Liang J., Wang L., Gao W., Jiang J., Chang R. (2020). Surfactin and fengycin B extracted from Bacillus pumilus W-7 provide protection against potato late blight via distinct and synergistic mechanisms. Appl. Microbiol. Biotechnol..

[56] Cadenas E., Davies K.J. (2000). Mitochondrial free radical generation, oxidative stress, and aging. Free Radic. Biol. Med..

[57] Jomova K., Raptova R., Alomar S.Y., Alwasel S.H., Nepovimova E., Kuca K., Valko M. (2023). Reactive oxygen species, toxicity, oxidative stress, and antioxidants: Chronic diseases and aging. Arch. Toxicol..

[58] Ayaz M., Ali Q., Zhao W., Chi Y.-K., Ali F., Rashid K.A., Cao S., He Y.-Q., Bukero A.A., Huang W.-K. (2024). Exploring plant growth promoting traits and biocontrol potential of new isolated *Bacillus subtilis* BS-2301 strain in suppressing *Sclerotinia sclerotiorum* through various mechanisms. Front. Plant Sci..

[59] Farzand A., Moosa A., Zubair M., Khan A.R., Hanif A., Tahir H.A.S., Gao X. (2019). Marker assisted detection and LC-MS analysis of antimicrobial compounds in different *Bacillus* strains and their antifungal effect on *Sclerotinia sclerotiorum*. Boil. Control.

[60] Pieterse C.M., Zamioudis C., Berendsen R.L., Weller D.M., Van Wees S.C., Bakker P.A. (2014). Induced systemic resistance by beneficial microbes. Annu. Rev. Phytopathol..

