# Supplementary material for: Biocontrol Mechanism of Bacillus thuringiensis GBAC46 Against Diseases and Pests Caused by Fusarium verticillioides and Spodoptera frugiperda
Source: Biomolecules. 2025 Apr 1;15(4):519. doi: 10.3390/biom15040519 (PMC12025000; doi:10.3390/biom15040519)
Supplement: Supplementary file 1 [file biomolecules-15-00519-s001.zip › biomolecules-3509054-supplementary.pdf]

Table S1 List strains used in this study

Strains	Characteristics	Source
GBAC46	*Bacillus thuringiensis*	Stored in our lab
NMTD81	*Bacillus thuringiensis*	Stored in our lab
FZB42	*Bacillus velezensis*	Stored in our lab
F. verticillioides	*Fusarium* spp.	Stored in our lab
F. oxysporum	*Fusarium* spp.	Stored in our lab
S. sclerotiorum	*Sclerotinia* spp.	Stored in our lab
R. solani	*Rhizoctonia* spp.	Stored in our lab

Table S2 Related genes for RT-qPCR and its primers used in this study

Genes	Primers	Sequence of primer (5′-3′)*
NPR1	Q-NPR1-F	ACCTTAATGAGACGCCAGTG
	Q-NPR1-R	CTCTTCACCTTCTGCTCATCG
PR1	Q-PR1-F	CTACGGCGAGAACCTCTTC
	Q-PR1-R	GTTGGTGTCGTGGTCGTAG
PR5	Q-PR5-F	TCCACAAGAAACAAGGCAGAG
	Q-PR5-R	TGTTGGTGATGGTGAAGGTG
AOS	Q-AOS -F	GACATGGACAAGGTGGAGAAG
	Q-AOS -R	GGAGAAGTTGGAGCGGAAG
MYC2	Q-MYC2- F	ATCGGGTCCACTTTTGAAGG
	Q-MYC2- R	CGAGGACAAATACAGGAAGGG
ERF	Q-ERF -F	GATGGCCTACGAGAACTACATG
	Q-ERF -R	CAGTAAAGAGCGACAGAGACG
CHIB	Q-CHIB -F	GTGGAACTACAACTACGGGC
	Q-CHIB -R	TTCATCCAGAACCAGAGCG
ACTIN	Q-Actin -F	GGTTCTATTCCAGCCATCCTTCATTG
	Q-Actin -R	TCTCCTTGCTCATGCGGTCAC
BX13	Q-BX13-F	CCTGCAATCTGAATTTCGAGTTC
	Q-BX13-R	TGAGTCACAAAGGTACCACAC
BX14	Q-BX14-F	GAGGGTGGGAAGGTGATAATC
	Q-BX14-R	CTGGTCGGATTTTGTAGTCTTTG
WIP	Q-WIP -F	CCGAGACCATGAAGAGCAG
	Q-WIP -R	AGAAGGAGAAGTTGCAGTTGG
LOX1	Q-LOX1-F	GCATTCCAAACAGCATCTCC
	Q-LOX1-R	AGAACCACACTTCACGGAAC
LOX5	Q-LOX5-F	GGATGCGTTCAAAAGATTCGG
	Q-LOX5-R	TGGAGATGCTGTTGGGAATG

TPS8	Q-TPS8-F	ACCAAGCTTCGATGACCATG
	Q-TPS8-R	TCTCTTGAAATCTGCCATGTCG
TPS10	Q-TPS10-F	GGGATGAACTAGGACCAAACAG
	Q-TPS10-R	ATGCTACTTGAAAGGCTCCC
ACO1	Q-ACO1-F	TCCATCGCATCCTTCTACAAC
	Q-ACO1-R	TTTATGCCGCTGGAGATGAC
Maize-Actin	Maize-actin-F	TACCATGTTCCCTGGGATTG
	Maize-actin-R	GTGGCGCAATCACTTTAACC
